# Chronic Breast Pain Prior to Breast Cancer Surgery Is Associated with Worse Acute Postoperative Pain Outcomes

**DOI:** 10.3390/jcm10091887

**Published:** 2021-04-27

**Authors:** Marium M. Raza, Ruth Zaslansky, Debra B. Gordon, Jeanne M. Wildisen, Marcus Komann, Ulrike M. Stamer, Dale J. Langford

**Affiliations:** 1 Department of Anesthesiology & Pain Medicine, University of Washington, Seattle, WA 98195, USA; mraza99@uw.edu (M.M.R.); debrag3@uw.edu (D.B.G.); 2 Department of Anesthesiology, University Hospital, 07747 Jena, Germany; ruth.zaslansky@med.uni-jena.de (R.Z.); Marcus.Komann@med.uni-jena.de (M.K.); 3 Department of Anesthesiology & Pain Medicine, Inselspital, Bern University Hospital, University of Bern, 3010 Bern, Switzerland; jeanne.wildisen@students.unibe.ch

**Keywords:** acute postoperative pain, breast cancer, preexisting chronic pain, patient-reported outcomes, pain registry, opioids

## Abstract

Acute postoperative pain is associated with adverse short and long-term outcomes among women undergoing surgery for breast cancer. Previous studies identified preexisting pain as a predictor of postoperative pain, but rarely accounted for pain location or chronicity. This study leveraged a multinational pain registry, PAIN OUT, to: (1) characterize patient subgroups based on preexisting chronic breast pain status and (2) determine the association of preexisting chronic pain with acute postoperative pain-related patient-reported outcomes and opioid consumption following breast cancer surgery. The primary outcome was a composite score comprising the mean of pain intensity and pain interference items from the International Pain Outcomes Questionnaire. The secondary outcome was opioid consumption in the recovery room and ward. Among 1889 patients, we characterized three subgroups: no preexisting chronic pain (*n* = 1600); chronic preexisting pain elsewhere (*n* = 128) and; chronic preexisting pain in the breast with/without pain elsewhere (*n* = 161). Controlling for covariates, women with preexisting chronic breast pain experienced more severe acute postoperative pain and pain interference (β = 1.0, 95% CI = 0.7-1.3, *p* < 0.001), and required higher doses of opioids postoperatively (β = 2.7, 95% CI = 0.6–4.8, *p* = 0.013). Preexisting chronic breast pain may be an important risk factor for poor pain-related postoperative outcomes. Targeted intervention of this subgroup may improve recovery.

## 1. Introduction

Breast cancer is the most common cancer diagnosis and the leading cause of disability-adjusted life years among women worldwide [1]. Most women (>90%) diagnosed with early stage breast cancer in the United States undergo surgery to remove the malignancy [2]. Nearly 60% experience severe acute pain following surgery for breast cancer [3], which is associated with increased morbidity and impaired quality of life [4,5]. Importantly, intensity of acute postoperative pain is associated with increased risk of chronic postsurgical breast pain [6,7,8,9,10] and prolonged opioid use [4,11,12]. Identifying patients at risk for severe postoperative pain will inform targeted and timely intervention efforts and practice patterns that may improve the postoperative pain experience and mitigate the risk of detrimental long-term outcomes.

Epidemiological studies and systematic literature reviews highlight preexisting pain as a robust predictor of postoperative pain. However, studies largely generalize across various surgical procedures (e.g., breast, orthopedic, general, gynecological, plastic, thoracic, vascular [13,14,15]), and do not specify the location and/or duration of preoperative pain. Theories explaining the association between pre- and postoperative pain have been posited, including that: (1) postoperative pain reflects a persisting presurgical condition [16], (2) preoperative pain reflects individual differences in pain sensitivity [17] or underlying biological differences (e.g., cytokine [18], potassium channel [19] gene variations) or (3) surgery-induced tissue/nerve injury overlaying preexisting pain leads to central sensitization [20]. Emerging evidence suggests that preexisting pain in the affected breast is a significant predictor of both acute postoperative [21,22] and chronic postsurgical breast pain among women undergoing breast cancer surgery [6,20,23], meriting further investigation.

Unlike other types of surgery (e.g., orthopedic), preexisting chronic pain—lasting more than three months [24]—at the site of surgery is not expected among women undergoing breast cancer surgery. As such, preexisting chronic breast pain is rarely investigated. One study was identified that found that chronic preoperative pain in the upper body was associated with significantly increased odds of severe movement-evoked pain in the acute postoperative period [20]. However, this study did not evaluate chronic breast pain as a predictor of postoperative pain, nor did it evaluate postoperative opioid consumption. A clearer understanding of preexisting breast pain as a risk factor for poorer pain and opioid outcomes will inform tailored interventions that may be implemented prior to surgery in order to improve recovery after breast cancer surgery.

This analysis of registry data leveraged a large, multinational database (PAIN OUT) to achieve the following objectives among women who underwent surgery for breast cancer: (1) characterize patient subgroups based on preexisting chronic breast pain status and (2) determine the association of preexisting chronic breast pain with acute postoperative pain outcomes and opioid consumption, accounting for covariates. Further, we utilized a global assessment of patient-reported outcomes (pain intensity, duration of severe pain, and pain-related functional interference) to capture multiple dimensions of the postoperative pain experience. 

## 2. Materials and Methods

### 2.1. Participants & Setting

We used data collected by PAIN OUT (http://pain-out.med.uni-jena.de/ accessed on 30 March 2021) to conduct this study. PAIN OUT is a multinational registry project that collects clinical and patient-reported data with the goal of improving pain care and postoperative pain outcomes (ClinicalTrials.gov: NCT02083835) [22]. Patients were eligible if they were: (1) 18 years of age or older, (2) at least six hours on the ward, and (3) gave consent. Patients were not eligible if they: (1) had cognitive impairments, (2) could not communicate, (3) were asleep, or (4) were too ill to participate. Local institutional review boards of participating institutions approved the study. The current analysis was exempt from IRB review at University of Washington. 

The PAIN OUT coordination team from the Department of Anesthesiology and Intensive Care Medicine at the University Hospital Jena (Germany) provided de-identified data from 2064 patients who underwent surgery for breast cancer between January 2011 and December 2018 from 62 participating institutions across 50 countries spanning Asia, Europe, North America, and Africa. Patients were included in this analysis if they had a documented diagnosis of breast cancer, were female, and provided complete data for the exposure (preexisting pain occurrence and location) and primary outcome (maximum acute postoperative pain intensity). 

### 2.2. Measures

Demographic and clinical data were extracted from medical records. Demographic variables included sex, age, height, weight and clinical site. Clinical variables included medical history, ICD-9 surgical procedure codes, and opioid, non-opioid and adjuvant analgesics throughout the perioperative process. Opioid dosages were recorded preoperatively, intraoperatively (preventative opioids), and postoperatively (recovery room and ward).

Patient-reported outcomes were collected using the validated International Pain Outcomes Questionnaire (IPOQ), which was adapted from the revised American Pain Society Patient Outcome Questionnaire [23,24]. The IPOQ and standard operating procedures are described in detail elsewhere [23,24]. The IPOQ was completed on postoperative day 1, reflecting approximately the first 24 h after surgery. Patients were asked to reflect on their pain experience since surgery. Pertinent to the current analyses, the IPOQ assessed:

◦ Pain intensity: Maximum (i.e., worst) and minimum (i.e., least) were rated on 0 “no pain” to 10 “worst pain imaginable” numeric rating scales (NRS) and the percent of time in severe pain since surgery (0% to 100%).
◦ Pain interference: Pain interference with activity in and out of bed, breathing deeply or coughing and sleeping, rated on a 0 “did not interfere” to 10 “completely interferes” NRS.
◦ Pain-related anxiety and helplessness: Anxiety and helplessness caused by pain rated on a 0 “not at all” to 10 “extremely” NRS.
◦ Perception of pain treatment: Pain relief was rated from 0 “no relief” to 100% “complete relief”. Patients were also asked to indicate they would have liked more pain treatment than they received (yes/no).
◦ Preexisting chronic pain: Patients were asked if they had preexisting pain for at least three months prior to surgery (yes/no), intensity of preexisting pain (0 to 10 NRS), and location of preexisting pain (surgical site [i.e., breast], elsewhere in the body, surgical site and elsewhere in the body).

### 2.3. Statistical Methods

We categorized patients into three subgroups based on occurrence and location of preexisting chronic pain: Group 0 = no preexisting chronic pain; Group 1 = preexisting chronic pain elsewhere in the body; Group 2 = preexisting chronic pain in the breast (surgical site) with or without pain elsewhere in the body. Our primary outcome of interest was a composite score of patient-reported outcomes (PRO) encompassing pain intensity and pain-related functional interference (i.e., PRO score) [25]. Our secondary outcome was postoperative opioid consumption in morphine equivalents (recovery room and ward).

Analyses were conducted using SPSS Version 26 [26]. We calculated frequency distributions and descriptive statistics to describe the study sample. We conducted one-way analyses of variance (ANOVAs) for continuous variables and chi square analyses for categorical variables, both with post hoc Bonferroni-corrected pairwise comparisons, to compare demographic, clinical, and pain characteristics among the subgroups. 

We conducted multivariable linear regression analyses to determine the association between preexisting chronic pain status and (1) the composite score of patient-reported outcomes (pain and pain-related interference and (2) postoperative opioid consumption (converted to morphine equivalents), controlling for key covariates. Preliminary multivariable regression models were fit with (1) demographic and clinical characteristics (age, BMI, continent, number of comorbidities, surgical factors and time since surgery) and (2) pre-admission and perioperative opioid dosages. Variables that remained statistically significantly (*p* < 0.05) associated with the outcome of interest from each of these models were included in the final adjusted model. To mitigate issues with heteroscedasticity, robust standard errors were employed to determine statistical significance in all regression models. 

## 3. Results

**Figure 1:**
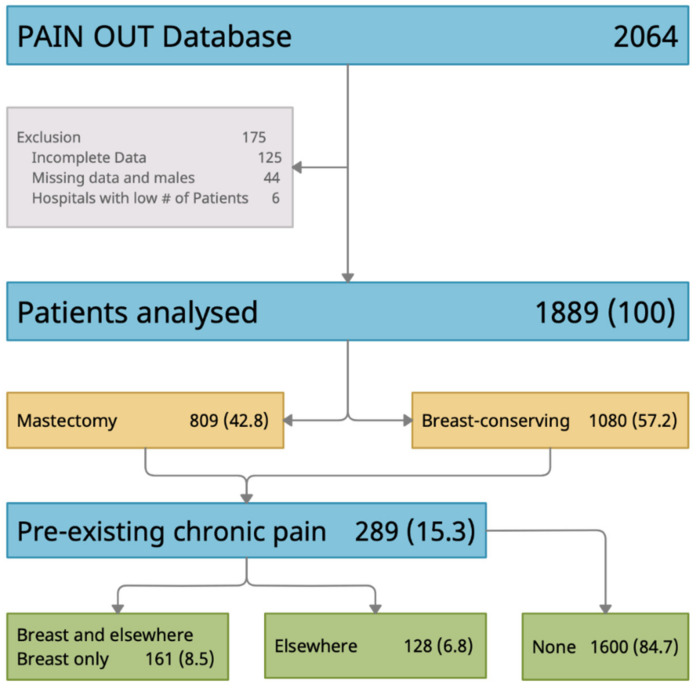
Flowchart describing analytic sample.

Data from 1889 women were included in the analyses (Figure 1) after excluding patients who were male (*n* = 37), gender was missing (*n* = 7), who did not provide data for occurrence of chronic preexisting pain (*n* = 118), location of preexisting pain (*n* = 6), or had discordant pain occurrence and location information (*n* = 1). In addition, women who underwent surgery at hospitals in Africa or North America were excluded due to the small sample sizes (*n* = 1 and *n* = 5, respectively).

### 3.1. Demographic and Clinical Characteristics

Table 1 describes the demographic and clinical characteristics for the total study sample and for each of the preexisting chronic pain subgroups. Of the total sample, 15.3% of patients reported chronic preexisting pain; 6.8% reported chronic pain elsewhere and 8.5% reported chronic pain in the breast with or without pain elsewhere in the body. On average, patients were 57 years old, most (80.9%) had at least one comorbidity, most commonly hypertension (36.1%). Further, 42.8% of patients underwent mastectomy (ICD9: 85.34, 85.4–85.48), 57.2% underwent breast conserving surgery (ICD9: 85.2–85.23). The average duration of surgical procedures was 97.6 min. Compared to women without preexisting chronic pain, patients with preexisting pain elsewhere (Group 1) were older and had a higher number of comorbidities. Women with chronic preexisting breast pain (Group 2) had a higher number of comorbidities than women without pain and were more likely to have an affective disorder compared with the both of the other subgroups.

### 3.2. Acute Postoperative Pain-Related Outcomes

Table 2 displays the means and 95% confidence intervals for pain-related outcomes among the subgroups. The composite pain intensity and interference variable differed significantly among the chronic preexisting pain subgroup, patients with chronic preexisting pain in the breast had higher scores than the other subgroups. Further, patients with breast pain had the highest scores on all of the variables comprising the composite score, as well as pain-related anxiety and helpless. We observed no significant difference among the subgroups in the amount of pain relief and a trend toward an increased proportion of patients who would have liked more pain treatment than they received.

### 3.3. Opioid Pain Management before Admission and Perioperatively

Table 3 provides information on the use of opioid analgesics for the total sample and each of the chronic preexisting pain subgroups before admission, intraoperatively (preventative) and postoperatively. Less than 2% of the total sample was taking opioids prior to admission. On average, patients with chronic preexisting pain elsewhere (Group 1) had significantly higher opioid MED before admission compared with the other subgroups. We observed no differences in intraoperative opioid MED among the subgroups. Postoperatively, patients with chronic preexisting pain in the breast (Group 2) had significantly higher opioid MEDs compared to patients with no preexisting pain.

### 3.4. Multivariable Linear Regression Findings

Table 4 displays findings from unadjusted and adjusted multivariable linear regression analyses with pain PRO-score as the outcome variable. Controlling for key covariates (see Supplemental Table S1 for preliminary regression models), preexisting chronic breast pain was associated with a 1-point increase in the composite score (95% CI: 0.66–1.33; *p* < 0.001; standardized B = 0.16) compared to women without chronic breast pain. Preoperative pain elsewhere was not significantly associated with a change in maximum postoperative pain intensity. Significant covariates in the final model included younger age (*p* < 0.001), mastectomy vs breast conserving surgery (*p* = 0.02), longer surgery duration (*p* = 0.021), receipt of regional anesthesia (*p* < 0.001) and higher postoperative opioid MED (*p* < 0.001). This final model accounted for 12.1% of the variance in composite pain intensity and interference outcome variable.

Table 5 displays unadjusted and final models for postoperative opioid MED as the outcome variable. Controlling for key covariates (see Supplementary Table S1), preexisting chronic pain was associated with a 3 mg increase in opioid consumption (95% CI: 0.6–4.8, *p* = 0.013; standardized B = 0.06). Chronic preexisting pain elsewhere was not associated with postoperative opioid dosage. Significant covariates in the final model included longer surgery duration (*p* < 0.001) and higher opioid MED before admission (*p* = 0.010). This final model accounted for 2.9% of the variance in postoperative opioid MED.

## 4. Discussion

We leveraged a unique, multinational data set, PAIN OUT, to study the association between preexisting chronic breast pain and pain-related patient reported outcomes and opioid consumption outcomes in women undergoing surgery for breast cancer. This study is unique in that subgroups of women were characterized based on preexisting chronic pain (rather than pain of any duration), as well as the location of their preexisting pain (i.e., elsewhere, breast +/− elsewhere). An additional strength of the study lies in the use of a multidimensional pain assessment and composite pain-related outcome score. Importantly, we demonstrated that preexisting chronic breast pain was associated with increased acute postoperative pain intensity, pain interference, and pain-related anxiety and helplessness, compared to patients with no chronic preoperative pain or preoperative chronic pain elsewhere in the body. In addition, preexisting chronic breast pain was associated with increased postoperative opioid consumption compared to those without preexisting pain.

In terms of demographic and clinical characteristics, patients with only chronic preexisting pain elsewhere in the body were particularly distinct. These patients were, on average, older than patients without chronic pain and had a higher number of comorbid conditions, particularly hypertension, compared with patients without chronic pain or with chronic pain in the breast. As such, preexisting chronic pain in this group was likely due to age-related events or other comorbid medical conditions.

Patients with preexisting chronic breast pain were characterized by a higher prevalence of comorbid affective disorders compared to patients without chronic pain. A higher prevalence of comorbid affective disorders is not surprising given the well-acknowledged interplay between chronic pain and psychological factors [25]. Further, it corroborates findings that preoperative breast pain is associated with increased mood disturbance in women scheduled for breast cancer surgery [18]. The finding is also in line with significantly elevated helplessness and anxiety ratings postoperatively among this subgroup of women. These findings highlight an association between chronic preexisting breast pain and psychological factors that, together with findings discussed below, characterize a subgroup at risk for poorer acute postoperative outcomes.

Patients with preexisting chronic pain in the breast had significantly higher acute postoperative pain intensity and interference composite scores compared to patients without preexisting pain and patients with preexisting chronic pain elsewhere. While the observed increase of ~1 point is small, an evaluation of Cohen’s d effect size (based on means, standard deviations and subgroup sample sizes) indicates a medium effect (d = 0.49). Further, a consistent pattern (Group 2 > 0 and 1) was observed among most of the items that comprise the composite score (i.e., least pain intensity, percent of time in severe pain since surgery, all pain interference items). 

Importantly, patients with preexisting chronic breast pain also required higher opioid dosages in the acute postoperative period compared to patients with no preexisting pain. This increase of ~3 mg is small (Cohen’s d effect size = 0.23) and therefore of questionable clinical significance. However, opioid doses were low and variability in opioid consumption was particularly high among women with preexisting breast pain compared to those without preexisting chronic pain (95% CIs of 5.1–9.3 vs 4.0–5.0), suggesting that while some patients had very little (if any) opioids postoperatively, others required relatively higher doses. Further, the postoperative opioid dosages overall (4.5 to 7.2 mg) were substantially lower than those reported in other similar samples (e.g., median 225–375 mg among > 4000 US patients) [26], and over half of the patients received no postoperative opioids at all, perhaps indicative of insufficient analgesia. 

Importantly, the association between preexisting breast pain and pain intensity/pain interference and postoperative opioid use remained significant in multivariable regression models, when controlling for covariates. Moreover, for both pain-related and opioid outcomes, preexisting breast pain appeared to have a relatively large effect (based on magnitude of standardized beta coefficient) compared to other variables in the model. Of note, preoperative pain intensity did not differ between women with pain in the breast (5.0 ± 2.6) and women with pain elsewhere (5.5 ± 2.6, *p* = 0.085, data not shown). Preexisting pain intensity therefore does not account for these subgroup differences. Rather, the repeated noxious stimulation to the breast (preexisting pain of unknown origin combined with surgical procedure) may offer a potential explanation for group differences. Collectively, these findings highlight the location of preexisting pain as a salient risk factor that warrants further investigation. 

Of note, the demographic and clinical covariates included in the models, while empirically determined, are supported by existing literature (e.g., younger age [17,22,27,28,29,30], surgery type [17,31], perioperative regional anesthesia [10,32]). It should be noted however, that the directionality of the relationship between postoperative opioid use and pain intensity/interference is unclear, as patients who experienced worse postoperative pain may have requested or required higher doses. 

Collectively, findings from this study indicate preexisting chronic pain as an independent and salient predictor of postoperative pain-related and opioid outcomes and may support the theory that postoperative pain reflect a persisting pre-surgical condition in the surgical area. Interestingly, 37 (23%) of patients with preexisting breast pain also reported chronic pain elsewhere in the body. Previous analyses of this small subgroup suggested a synergistic effect, possibly suggestive of underlying central sensitization [20]. Increased awareness of and surveillance for preexisting breast pain is necessary. Furthermore, a better understanding of the cause of pain (e.g., disease-related, insidious) may be critical to inform intervention efforts. Importantly, a recent study highlighted the importance of a regimented perioperative protocol—for pain management for enhanced recovery after surgery. While this sample included 31.7% with preoperative chronic pain problems, only 13% had chronic postsurgical pain at one year [10].

Limitations of this study should be noted. The prevalence of patients with chronic preexisting breast pain is low (8.5% of the total sample), and therefore clinical implications may be limited. However, given that the IPOQ asks about chronic preoperative pain and not preoperative pain of any duration, this study likely underestimates the prevalence of preexisting pain (of any duration). In fact, other studies of preexisting breast pain have reported estimates of ~30%, and demonstrated a significant association with acute and chronic pain outcomes [22]. Furthermore, we were unable to determine the etiology of preexisting chronic pain (in the breast or elsewhere) based on PAIN OUT. Data collection was limited to postoperative day one, precluding the evaluation of preoperative breast pain as a predictor of longer-term clinical outcomes. However, longitudinal data exist for a subset of the sample and may be the focus of future analyses. Importantly, because PAIN OUT spans all types of surgery, we do not have specific disease-related information (e.g., stage of disease, lymph node involvement, metastasis). Likewise, the pain interference items may not adequately reflect interference from breast cancer surgery in particular (e.g., upper body function). Finally, the adjusted model accounted for only 12% of the variance in PRO scores, suggesting that parameters other than those collected are relevant for acute postoperative pain. For example, psychological factors, such as anxiety, pain catastrophizing or anticipation of pain may play an important role [33,34]. In addition, cultural factors and differences in healthcare environments across participating countries may contribute to variance in pain-related outcomes [35].

## 5. Conclusions

Overall, this study demonstrates the utility of a large pain outcome registry to characterize a unique and salient predictor of pain-related and opioid analgesic outcomes in women undergoing surgery for breast cancer. Preexisting chronic breast pain may be an important risk factor for postoperative pain that merits further investigation. The preoperative identification and targeted intervention of these patients may improve acute postoperative pain-related outcomes and enhance the recovery trajectory. 

## Figures and Tables

**Table 1:** Demographic and clinical characteristics of total sample and preexisting chronic pain subgroups.

	Total Sample *N* = 1889	No Chronic Pain Group 0 *n* = 1600 (84.7%)	Pain Elsewhere Group 1 n = 128 (6.8%)	Pain in Breast * Group 2 *n* = 161 (8.5%)	Statistics Pairwise Post Hoc Contrasts
	Mean (95% CI)	Mean (95% CI)	Mean (95% CI)	Mean (95% CI)	
Age	56.6 (56.0–57.3)	56.2 (55.6–56.9)	62.1 (59.9–64.3)	56.5 (54.1–58.90	F = 10.9, *p* < 0.001 ** 1 > 0 and 2
Body Mass Index	26.4 (26.2–26.7)	26.3 (26.0–26.6)	27.2 (26.2–28.3)	27.5 (26.4–28.6)	F = 4.0, *p* = 0.019 No sig pw contrasts
# of Comorbidities	1.6 (1.6–1.6)	1.5 (1.5–1.6)	1.9 (1.7, 2.1)	1.9 (1.7, 2.1)	F = 13.2, *p* < 0.001 0 < 1 and 2
	**n (%)**	**n (%)**	**n (%)**	**n (%)**	
Any comorbidity	1304 (80.9)	1091 (80.3)	108 (91.5)	105 (77.8)	X^2^ = 9.8, *p*= 0.008 1 > 0 and 2
Hypertension	476 (36.1)	389 (35.3)	52 (47.7)	35 (33.0)	X^2^ = 7.1, *p* = 0.028 1 > 0
Smoker	156 (11.8)	130 (11.8)	10 (9.2)	16 (15.1)	X^2^ = 1.8, *p* = 0.402
Affective disorder	94 (7.1)	70 (6.3)	8 (7.3)	16 (15.1)	X^2^ = 11.2, *p* = 0.004 2 > 0
Asthma	61 (4.6)	44 (4.0)	8 (7.3)	9 (8.5)	X^2^ = 6.4, *p* = 0.040 No sig pw contrasts
Coronary disease	56 (4.2)	42 (3.8)	8 (7.3)	6 (5.7)	X^2^ = 3.6, *p* = 0.165
Surgery Mastectomy Breast Conserving	809 (42.8) 1080 (57.2)	679 (42.4) 921 (57.6)	54 (42.2) 74 (57.8)	76 (47.2) 85 (52.8)	X^2^ = 1.4, *p* = 0.501
	**Mean (95% CI)**	**Mean (95% CI)**	**Mean (95% CI)**	**Mean (95% CI)**	
Surgery duration (min)	97.6 (94.9–100.3)	97.6 (94.7–100.5)	94.6 (86.6–102.55)	103.3 (93.7–113.0)	X^2^ = 0.9, *p* = 0.395

* Group 2 had preexisting chronic pain in the breast with or without chronic pain elsewhere (group name abbreviated for clarity). ** Omnibus *p*-values not corrected for multiple comparisons. Post hoc contrasts are Bonferroni corrected.

**Table 2:** Acute postoperative pain-related outcomes among preexisting chronic pain subgroups.

Pain Outcome	Total Sample *N* = 1889	No Chronic Pain Group 0 *n* = 1600 (84.7%)	Pain Elsewhere Group 1 *n* = 128 (6.8%)	Pain in Breast Group 2 *n* = 161 (8.5%)	Statistics Pairwise Post Hoc Contrasts
	M (95% CI)	M (95% CI)	M (95% CI)	M (95% CI)	
Pain Intensity + Interference Composite Score	2.4 (2.3–2.5)	2.3 (2.2–2.4)	2.3 (2.0–2.5)	3.3 (2.9–3.6)	F = 20.8, *p* < 0.001 2 > 0 and 1
Worst Pain Intensity	4.0 (3.8–4.1)	3.9 (3.7–4.0)	4.2 (3.7–4.6)	4.9 (4.4–5.4)	F = 11.9, *p* < 0.001 2 > 0
Least Pain Intensity	1.4 (1.3–1.5)	1.3 (1.2–.4)	1.2 (1.0–1.5)	2.1 (1.8–2.5)	F = 20.0, *p* < 0.001 2 > 0 and 1
% Time in Severe Pain since Surgery	18.1 (17.2–19.0)	17.4 (16.5–18.4)	16.8 (13.6–9.9)	26.2 (23.4–30.1)	F = 14.5, *p* < 0.001 2 > 0 and 1
Pain Interference with Activities in Bed Pain Interference with Breathing Deeply/Coughing Pain Interference with Sleeping Pain Interference with Activities Out of Bed	3.3 (2.2–3.4) 1.4 (1.3–1.6) 1.9 (1.8–2.0) 2.1 (2.0–2.2)	3.2 (3.1–3.4) 1.4 (1.3–1.5) 1.8 (1.7–2.0) 2.0 (1.9–2.1)	3.4 (2.9–3.9) 1.3 (0.9–1.7) 1.9 (1.5–2.3) 2.2 (1.7–2.7)	4.6 (4.1–5.0) 2.3 (2.2–3.2) 2.7 (2.2–3.2) 3.3 (2.8–3.7)	F = 16.6, *p* < 0.001 2 > 0 and 1 F = 10.6, *p* < 0.001 2 > 0 and 1 F = 7.6, *p* = 0.001 2 > 0 and 1 F =20.4, *p* < 0.001 2 > 0 and 1
Pain-Related Anxiety	1.7 (1.6–1.8)	1.6 (1.5–1.7)	1.6 (1.2–2.1)	2.9 (1.8–2.8)	F = 22.1, *p* < 0.001 2 > 0 and 1
Pain-Related Helplessness	1.4 (1.3–1.5)	1.3 (1.2–1.4)	1.5 (1.1–2.0)	2.3 (1.8–2.8)	F = 12.4, *p* < 0.001 2 > 0 and 1
Pain Relief (%)	73.9 (72.7–75.2)	74.0 (72.6–75.4)	74.7 (70.0–79.5)	72.6 (68.3–76.9)	F = 0.2, *p* = 0.784
	**n (%)**	**n (%)**	**n (%)**	**n (%)**	
Would have liked more pain treatment than received	154 (8.3%)	127 (8.1%)	7 (5.5%)	20 (12.6%)	X^2^ = 5.2, *p* = 0.073

**Table 3:** Opioid analgesic use before admission and perioperatively *.

Opioids	Total Sample *N* = 1889	No Chronic Pain Group 0 *n* = 1600 (84.7%)	Pain Elsewhere Group 1 *n* = 128 (6.8%)	Pain in Breast Group 2 *n* = 161 (8.5%)	Statistics Pairwise Post Hoc Contrasts
Before Admission MED *n* (%) Mean (95% CI)	32 (1.7) 0.6 (0.2–1.0)	12 (0.8) 0.2 (0.03–0.3)	13 (10.4) 4.5 (−1.2–10.2)	7 (4.6) 1.6 (−0.1–3.4)	F = 13.8, *p* < 0.001 1 > 0 and 2
Intraoperative MED *n* (%) Mean (95% CI)	559 (29.6) 2.2 (1.6–2.7)	478 (29.9) 2.2 (1.6–2.8)	39 (30.5) 2.3 (1.6–2.9)	42 (26.1) 1.7 (1.2–2.3)	F = 0.1, *p* = 0.895
Postoperative MED *n* (%) Mean (95% CI)	816 (43.2) 4.9 (4.3–5.4)	679 (42.5) 4.5 (4.0–5.0)	59 (46.1) 6.1 (2.0–10.1)	78 (48.4) 7.2 (5.1–9.3)	F = 4.7, *p* = 0.009 2 > 0

* Note: Descriptive statistics include 0 mg dosages for patients not taking opioids. The number of patients taking opioids at each phase is included for clarification. Abbreviations: CI = confidence interval; MED = morphine equivalent dosage.

**Table 4:** Unadjusted and adjusted multiple linear regression models with outcome of composite pain and pain interference (PRO) score on postoperative day one.

Unadjusted Model (*n* = 1884)/F = 20.8, *p* < 0.001 (R^2^ = 0.022)							
	B	Robust SE	Std. B	t	p	95% Confidence Interval	
Preexisting Chronic Pain							
No Chronic Pain	(ref)	-	-	-	-	-	-
Pain Elsewhere	0.02	0.15	0.002	0.11	0.914	−0.28	0.32
Pain in Breast	0.96	0.18	0.15	5.36	<0.001	0.61	1.31
**Adjusted Model (*n* = 1285)/F=19.6, *p* < 0.001 (R^2^ = 0.121)**							
	**B**	**Robust** **SE**	**Std. B**	**t**	**p**	**95% Confidence Interval**	
Preexisting Chronic Pain							
No Chronic Pain	(ref)	-	-	-	-	-	-
Pain Elsewhere	0.08	0.16	0.01	0.51	0.611	−0.23	0.39
Pain in Breast	1.00	0.22	0.16	4.62	<0.001	0.58	1.44
Age (years)	−0.03	0.004	−0.20	−6.59	<0.001	−0.04	−0.02
Number of Comorbidities	0.09	0.06	0.05	1.56	0.118	−0.02	0.21
Region (Europe)	0.20	0.15	0.07	1.32	0.198	−0.10	0.50
Surgery Type (mastectomy)	0.23	0.10	0.07	2.20	0.028	0.02	0.43
Surgery Duration (min)	0.002	0.001	−0.11	2.32	0.021	0.000	0.004
Intraop Regional Anesthesia (yes)	−0.82	0.18	0.04	−4.67	<0.001	−1.17	−0.48
Postop Opioid MED (mg)	0.02	0.009	0.15	2.32	0.02	0.03	0.04

Abbreviations: B = Beta coefficient; MED = morphine equivalent dose; mg = milligrams; min = minute; ref = reference group; SE = standard error; Std = standardized.

**Table 5:** Unadjusted and adjusted multiple linear regression models with outcome of opioid morphine equivalent dosage in first 24 h after surgery (recovery room and ward).

Unadjusted Model (*n* = 1889)/F = 4.702, *p* = 0.009 (R^2^ = 0.005)							
	B	Robust SE	Std. B	t	p	95% Confidence Interval	
Preexisting Chronic Pain							
No Chronic Pain	(ref)	-	-	-	-	-	-
Pain Elsewhere	1.55	2.06	0.03	0.75	0.452	−2.49	5.58
Pain in Breast	2.67	1.10	0.07	2.44	0.015	0.52	4.82
**Adjusted Model (*n* = 1564)/F = 8.33, *p* < 0.001 (R^2^ = 0.026)**							
	**B**	**Robust** **SE**	**Std. B**	**t**	**p**	**95% Confidence Interval**	
Preexisting Chronic Pain							
No Chronic Pain	(ref)	-	-	-	-	-	-
Pain Elsewhere	2.09	2.31	0.05	0.90	0.366	−2.44	6.63
Pain in Breast	2.56	1.22	0.06	2.11	0.035	0.18	4.95
Age (years)	−0.04	0.02	−0.05	−2.48	0.013	−0.08	−0.009
Surgery Duration (min)	0.03	0.006	0.12	4.21	<0.001	0.01	0.04
Intraop Regional Anesthesia (yes)	0.08	0.03	−0.05	2.57	0.010	0.02	0.13

Abbreviations: B = Beta coefficient; Intraop = intraoperative; MED = morphine equivalent dose; mg = milligrams; min = minutes ref = reference group; SE = standard error; Std. = standardized.

## Data Availability

Restrictions apply to the availability of these data. Data were obtained from the PAIN OUT registry upon approval of publication proposal. More details can be found at http://pain-out.med.uni-jena.de/publications.

## Supplementary Materials

The following are available online at https://www.mdpi.com/article/10.3390/jcm10091887/s1, Table S1: Preliminary multiple linear regression models for outcomes of (1) composite pain and pain interference score and (2) postoperative opioid MED (in recovery room and on ward).
