# Supplementary material for: Chronic Breast Pain Prior to Breast Cancer Surgery Is Associated with Worse Acute Postoperative Pain Outcomes
Source: J Clin Med. 2021 Apr 27;10(9):1887. doi: 10.3390/jcm10091887 (PMC8123777; doi:10.3390/jcm10091887)
Supplement: Supplementary file 1 [file jcm-10-01887-s001.zip › jcm-1185096-supplementary.pdf]

Supplementary Table S1. Preliminary multiple linear regression models for outcomes of (1) composite pain and pain interference score and (2) postoperative opioid MED (in recovery room and on ward). Separate models were conducted for (a) demographic and clinical and (b) pre-admission and perioperative opioid MED independent variables.

**1a) Outcome: Pain and Pain Interference Composite Score<br>Demographic & Clinical Variables (*n* = 1104) / F = 12.7, *p* < 0.001 (R^2 = 0.094)**						
	**B**	**Robust SE**	**t**	**P>t**	**95% Confidence Interval**	
Age (years)	−0.03	0.004	−7.08	<0.001	−0.04	−0.02
Body Mass Index	0.001	0.009	0.07	0.947	−0.02	0.02
Number of Comorbidities	0.14	0.07	2.12	0.034	0.01	0.27
Region (Europe)	0.44	0.17	2.63	0.009	0.11	0.77
Surgery Type (mastectomy)	0.36	0.12	3.06	0.002	0.13	0.59
Axillary Lymph Node Dissection (yes)	−0.12	0.14	−0.91	0.365	−0.39	0.14
Surgery Duration (min)	0.003	0.001	3.26	0.001	0.001	0.006
Intraop Regional Anesthesia (yes)	−0.80	0.20	−4.09	<0.001	−1.18	−0.42
Duration between Surgery and IPOQ (hours)	0.01	0.01	1.11	0.267	−0.01	0.04
**1b) Outcome: Pain and Pain Interference Composite Score<br>Pre-Admission and Perioperative Opioid MED (*n* = 1876)/F = 10.7, *p* < 0.001 (R^2 = 0.017)**						
	**B**	**SE**	**t**	**P>t**	**95% Confidence Interval**	
Pre-Admission Opioid MED (mg)	0.007	0.01	0.47	0.640	−0.02	0.04
Intraoperative Opioid MED (mg)	0.000	0.002	−0.02	0.981	−0.003	0.003
Postop Opioid MED	0.02	0.006	3.18	0.001	0.01	0.03

**2a) Outcome: Postoperative Opioid MED<br>Demographic & Clinical Variables (*n* = 1105)/F = 2.91, p = 0.002 (R^2 = 0.023)**						
	**B**	**Robust SE**	**t**	**P>t**	**95% Confidence Interval**	
Age (years)	−0.07	0.02	−3.59	<0.001	−0.11	−0.03
Body Mass Index	0.09	0.07	1.35	0.177	−0.04	0.22
Number of Comorbidities	−0.12	0.32	−0.37	0.708	−0.74	0.50
Region (Europe)	−0.78	1.8	−0.44	0.663	−4.30	2.74
Surgery Type (mastectomy)	0.22	0.88	0.25	0.804	−1.50	1.94
Axillary Lymph Node Dissection (yes)	1.20	0.84	1.43	0.153	−0.45	2.89
Surgery Duration (min)	0.02	0.006	2.78	0.005	0.005	0.03
Intraop Regional Anesthesia (yes)	−2.17	0.87	−2.49	0.013	−3.87	0.46
Duration between Surgery and IPOQ (hours)	0.02	0.12	0.17	0.865	−0.22	0.26
**2b) Outcome: Postoperative Opioid MED<br>Pre-Admission and Perioperative Opioid MED (*n* = 1881)/F = 5.01, *p* = 0.007 (R^2 = 0.005)**						
	**B**	**Robust SE**	**t**	**P>t**	**95% Confidence Interval**	
Pre-Admission Opioid MED (mg)	0.09	0.34	0.26	0.792	−0.58	0.76
Intraoperative Opioid MED (mg)	0.000	0.04	−0.001	0.999	−0.09	0.09

Abbreviations: B = Beta coefficient; mg = milligrams; min = minutes; SE = standard error
